# IMPA2 ameliorates high-fat diet-induced obesity by enhancing adaptive thermogenesis in a Ca²⁺-activated mitochondrial biogenesis pathway

**DOI:** 10.1186/s12967-026-08592-2

**Published:** 2026-07-08

**Authors:** Li Lin, Yudan Zhou, Lulu Ning, Ruiqi Mao, Yuhang Wang, Meng Du, Le Chen, Kai Huang, Min Chen

**Affiliations:** 1 Clinic Center of Human Gene Research, Union Hospital, Tongji Medical College, Huazhong University of Science and Technology, Wuhan, China; 2 Hubei Key Laboratory of Metabolic Abnormalities and Vascular Aging, Huazhong University of Science and Technology, Wuhan, China; 3 Hubei Clinical Research Center of Metabolic and Cardiovascular Disease, Huazhong University of Science and Technology, Wuhan, China; 4 Department of Cardiology, Union Hospital, Tongji Medical College, Huazhong University of Science and Technology, Wuhan, China; 5 Department of Cardiology, Liyuan Hospital, Tongji Medical College, Huazhong University of Science and Technology, Wuhan, China; 6 Department of Pathophysiology, School of Basic Medicine, Tongji Medical College, Huazhong University of Science and Technology, Wuhan, China; 7 Department of Obstetrics and Gynecology, Union Hospital, Tongji Medical College, Huazhong University of Science and Technology, Wuhan, China; 8 Department of Endocrinology, Union Hospital, Tongji Medical College, Huazhong University of Science and Technology, Wuhan, China

## Abstract

**Background:**

Obesity poses a significant threat to global human health. The identification of thermogenic adipocytes in humans proves that enhanced adaptive thermogenesis may help mitigate obesity. Although some evidence suggests a role for IMPA2 in cellular metabolism, its specific impact on obesity and the underlying mechanisms remain underexplored.

**Methods:**

High-fat-diet (HFD)-induced obese mice were employed as experimental models. Cold exposure (4 °C) or administration of the β3-adrenoceptor agonist CL316,243 was used to stimulate adipose thermogenesis. In vivo modulation of IMPA2 function was achieved through subcutaneous injection of adeno-associated virus (AAV). Body weight and energy metabolism were monitored by the CLAMS, and browning was determined. The role and mechanisms of IMPA2 in thermogenesis were examined through adenovirus (Adv) treatment, followed by in vitro and in vivo analyses.

**Results:**

Thermogenic stimulation increased IMPA2 level in murine inguinal white adipose tissue (iWAT), whereas obesity resulted in its downregulation. IMPA2 overexpression in iWAT accelerated iWAT browning and thermogenesis, conferring protection against HFD-induced obesity and metabolic disturbances. Conversely, silencing IMPA2 reduced thermogenic gene levels in iWAT and inhibited WAT browning. Mechanistically, IMPA2 elevated myo-inositol (MI), inositol-1,4,5-trisphosphate (IP3) and intracellular calcium ion (Ca²⁺) levels, which subsequently activated Ca²⁺-dependent protein kinase IIα (CamKIIα) and PGC1α axis to increase mitochondrial biogenesis and thermogenic capacity in adipocytes.

**Conclusions:**

IMPA2 plays a crucial role in adaptive thermogenesis and energy expenditure. IMPA2 improves obesity and metabolic disorders, with its thermogenic enhancement mediated by the Ca²⁺-activated mitochondrial biogenesis pathway.

**Supplementary Information:**

The online version contains supplementary material available at https://doi.org/10.1186/s12967-026-08592-2.

## Introduction

Beige adipocytes (also known as inducible brown, brown-in-white, or brite adipocytes), a recently identified subtype of adipocytes, emerge within white adipose tissue (WAT) under thermogenic stimuli, a phenomenon described as the “browning” of WAT [1, 2]. Major inducers of browning include cold exposure and β3-adrenoceptor agonist stimulation [3, 4]. Beige adipocytes share several characteristics with brown adipocytes, encompassing a higher mitochondrial content, production of mitochondrial uncoupling protein 1 (UCP1) in the inner mitochondrial membrane, and multilocular lipid droplets, distinguishing them from white adipocytes [5–7]. These cells not only increase energy expenditure but also improve glucose clearance, reduce fibrosis, and confer significant benefits to metabolic health [8–10]. Multiple investigations have suggested that enhanced levels or function of beige adipocytes facilitates a lean and healthy metabolic phenotype [11, 12]. In contrast, reduced beige adipocyte activity is linked to obesity and metabolic dysfunction, contributing to insulin resistance, type 2 diabetes mellitus, and cardiometabolic diseases [3, 13]. Although the UCP1-dependent thermogenic pathway is widely understood, recent studies have reported the discovery of a UCP1-independent thermogenic pathway in thermogenic adipocytes [14, 15]. The UCP1-independent thermogenic pathway relies on enhanced ineffective calcium and creatine cycles to consume ATP for heat production, whereas the UCP1-dependent thermogenic pathway consumes energy in the form of heat by uncoupling the mitochondrial proton gradient from mitochondrial respiration [16, 17]. Therefore, elucidating the molecular mechanisms involved in regulating WAT browning and thermogenesis holds substantial importance for developing treatment approaches to address obesity-associated metabolic conditions.

IMPA2 (inositol(myo)-1(or 4)-monophosphatase 2), a promising gene situated on the short arm of chromosome 18 at position 11.2 (18p11.2) [18], encodes an enzyme involved in inositol metabolism and cell signaling [19]. IMPA2 catalyzes the production of inositol, a molecule essential for various cellular processes, including osmoregulation, cell growth, and signal transduction. It exhibits broad substrate specificity, including inositol monophosphate, scyllo-inositol 1,4-bisphosphate, glucose-1-phosphate, β-glycerophosphate, and 2’-AMP, highlighting its role in the hydrolysis of inositol-containing phosphates [20, 21]. The IMPA2 enzyme can bind metal ions, including magnesium (Mg²⁺), lithium (Li⁺), and calcium (Ca²⁺), with two Ca²⁺ occupying the active site of the monomer [22]. IMPA2 has been implicated in various neuropsychiatric disorders, including bipolar disorder, schizophrenia, and febrile seizures [23–26]. Reduced IMPA2 functionality and heightened baseline intracellular Ca²⁺ concentrations were detected in B lymphoblast cell lines (BLCLs) derived from patients with bipolar I affective disorder (BD-I) [26]. Notably, IMPA2 serves as a pharmacological target for Li^+^ in the brain, highlighting its potential role in regulating intracellular inositol levels and emphasizing its contribution to lithium’s therapeutic effects [27, 28]. In the realm of cancer, IMPA2 has been implicated in various malignancies, including basal-like breast cancer (BLBC), cervical cancer and non-small cell lung cancer [21, 29–34]. Studies have demonstrated that downregulation of IMPA2 elevates mTORC1 function and blocks autophagy commencement in metastatic clear cell renal cell carcinoma [35]. In BLBC, IMPA2 levels are markedly elevated compared to other subtypes, where it promotes cell aggressiveness by facilitating the Myo-inositol (MI)-Phosphatidylinositol (PI) cycle and increasing inositol-1,4,5-triphosphate (IP3) generation. This, in turn, raises intracellular Ca²⁺ concentrations, triggering efficient activation of NFAT1-MYC expression [29]. In summary, IMPA2’s role in inositol metabolism, cell signaling, and lithium therapy underscores its importance in cellular processes.

Ca²⁺ serves a pivotal function in various cellular functions, including muscle contraction, blood coagulation, gene transcription, apoptosis, fertilization, and cellular metabolism [36, 37]. Intracellular Ca²⁺ levels are precisely controlled through diverse Ca²⁺ channels and pumps localized at the endoplasmic reticulum (ER) membrane [38]. Research has shown that Ca²⁺ is integral to adipocyte thermogenesis [39–42]. Ikeda and colleagues identified that Ca²⁺ cycling-mediated heat production serves as a UCP1-independent thermogenic mechanism in beige adipocytes. The ATP-dependent Ca²⁺ uptake channel, SERCA2b, along with Ca²⁺ release channels, such as RyR2 and IP3 receptor (IP3R), work in concert to maintain Ca²⁺ homeostasis [39]. IP3 triggers Ca²⁺ release from the ER *via* the IP3R embedded in the ER membrane [43]. Another study found that loss of folliculin interacting protein 1 (FNIP1) led to enhanced intracellular Ca²⁺ signaling, which activated the Ca²⁺-dependent thermogenic pathways in adipocytes. Mice with FNIP1-deficient adipocytes exhibited resistance to HFD-induced insulin resistance and liver steatosis [40, 44]. In addition, Ca²⁺-dependent kinases have been shown to activate transcriptional reprogramming of mitochondrial biogenesis and metabolism *via* PGC1α, and mitochondrial function is an important influence on the thermogenic capacity of adipocytes [45, 46]. Adrenergic stimulation of thermoporter formation increases Ca²⁺ uptake in mitochondria and proton availability, thereby promoting thermogenesis [42]. Given its role in inositol metabolism, which is closely linked to insulin sensitivity and glucose homeostasis [47], IMPA2 activity may markedly influence these processes. Disruptions in inositol metabolism could lead to insulin resistance, a key contributor to obesity and related metabolic disorders, although no studies have yet linked IMPA2 to obesity.

In this study, the metabolic function of IMPA2 signaling in thermogenic adipocytes was investigated in mice. IMPA2 was identified through the analysis of two datasets (GSE86338 and GSE164219), where its expression was found to increase under cold conditions (Fig. S1). Adipocyte-specific IMPA2 overexpression and knockout mice were generated, revealing that IMPA2 functions as a positive modulator of beige adipocyte thermogenesis, thereby influencing systemic glucose homeostasis. We observed that IMPA2 expression in inguinal white adipose tissue (iWAT) was upregulated by thermogenic stimuli (cold exposure or β3-adrenoceptor agonist) and downregulated in obesity. Further analysis demonstrated that adipocyte-specific overexpression of IMPA2 accelerated WAT browning and shielded mice against HFD-induced glucose intolerance, insulin resistance, and liver steatosis. Conversely, adipocyte-specific ablation of IMPA2 resulted in reduced thermogenic activity in adipose tissue. Mechanistically, IMPA2 enhances intracellular MI, IP3 and Ca²⁺ levels, subsequently activating Ca²⁺-dependent protein kinase II (CamKIIα) and PGC1α axis to increase mitochondrial biogenesis and thermogenic capacity in adipocytes. Furthermore, the role of IMPA2 in promoting calcium-induced thermogenesis in adipocytes does not depend on thermogenic independent UCP1 pathway involving SERCA2b-mediated calcium cycling. These findings reveal a previously unrecognized IMPA2-intracellular Ca²⁺-activated mitochondrial biogenesis pathway that regulates WAT browning.

## Materials and methods

### Animal studies

Wild-type (WT) C57BL/6J male mice and db/db male mice, aged 6 or 8 weeks, were purchased from Weitong Lihua Co., Ltd. (Beijing, China) and used for experiments. Animals were assigned to experimental groups using simple randomization. All mice were pathogen-free and housed in ventilated isolation cages under controlled conditions: 30 °C room temperature, 40–60% humidity, unrestricted water and food availability, and a 12-hour light/12-hour dark cycle. For in vivo studies involving iWAT-IMPA2 upregulation and suppression, adeno-associated virus 9 (AAV9) containing IMPA2 (AAV-FABP4-IMPA2) or short hairpin RNA (shRNA) targeting IMPA2 (AAV-FABP4-shIMPA2) was subcutaneously injected into the bilateral inguinal regions. AAV9 carrying control (AAV-FABP4-NC) or scramble shRNA (AAV-FABP4-shNC) served as controls. For HFD-induced obesity, mice were fed either a HFD (60% of total kilocalories from fat, D12492) for 12 weeks or a standard control diet (SCD) for 12 weeks, beginning 2 weeks after AAV injection, with body weight monitored weekly. For the cold exposure experiment, mice underwent a 48-hour exposure to 4 °C following two weeks after AAV administration. In the CL316,243 experiment, mice were administered daily intraperitoneal injections of either saline or CL316,243 (1 mg/kg/d) for 5 days. Animals with incomplete lesions were excluded from the study. All animals completed the study with no dropouts. All analyses were performed in a blinded manner, such that neither the experimenters nor the data analysts were aware of the genotypes and treatments. All animal protocols were sanctioned by the Institutional Animal Care and Use Committee of Tongji Medical College, Huazhong University of Science and Technology, and all procedures were performed in compliance with the ARRIVE protocols.

### Glucose and insulin tolerance tests (GTT and ITT)

Glucose tolerance tests (GTT) were conducted after a 16-hour fast, with subsequent intraperitoneal administration of glucose (2 g/kg, Solarbio, China). One week after the GTT, mice received insulin injections (0.75 unit/kg, Biosharp, China) following a 4-hour fast. Blood glucose levels were monitored at intervals of 0, 15, 30, 60, 90, and 120 min following the administration of either glucose or insulin utilizing a glucose meter.

### Blood analysis

Serum concentrations of low-density lipoprotein cholesterol (LDL), high-density lipoprotein cholesterol (HDL), total cholesterol (TC), and triglycerides (TG) were quantified utilizing chemical reagent assay kits (LDL assay kit, A113-1-1; HDL assay kit, A112-1-1; TC assay kit, A111-1-1; TG assay kit, A110-1-1, Nanjing Jian Cheng Bioengineering Institute, China) per the supplier’s protocols. Insulin levels in serum were assessed utilizing commercial enzyme-linked immunosorbent assay (ELISA) kits (PI602, Beyotime, China). TNF-α and IL-6 levels in serum were assessed utilizing commercial enzyme-linked immunosorbent assay (ELISA) kits (E-HSEL-M0009, Elabscience, China).

### Metabolic analysis

Energy expenditure was measured by individually housing mice in metabolic cages linked to the Oxymax-Comprehensive Laboratory Animal Monitoring System (Oxymax-CLAMS, Columbus Instruments, USA) at 30 °C–4 °C, under a 12-hour light/dark cycle. Mice had free access to food and water. Each mouse was continuously monitored for physical activity and food intake. CO_2_ and O_2_ levels were collected over the duration of the experiment.

### Cold exposure and temperature measurement

Two weeks post-virus injection, mice were placed in 4 °C environments in individual cages, and rectal temperatures were recorded at 0, 1, 2, 4, and 6 h.

### Virus preparation

Adipose-specific overexpression or knockdown of target genes was achieved using adeno-associated virus serotype 9 (AAV9) driven by the fatty acid-binding protein 4 (FABP4) promoter. General Biol (Anhui) Co., Ltd., constructed AAV9-FABP4-IMPA2 and AAV9-FABP4-Sh-IMPA2, along with a corresponding control AAV9-FABP4-Ctrl. Recombinant AAV viruses were used for in vivo transduction of iWAT, while adenoviruses were utilized to infect primary adipose cells in vitro. The sequence of mouse sh-IMPA2 is as follows: 5′-CACCGAGTAGAAGATTTAATT-3′. All viral constructs were provided by General Biol (Anhui) Co. Ltd.

### Isolation of stromal vascular fraction (SVF) and mature adipocytes

SVF cells and mature adipocytes were isolated from 8-week-old male mice following exposure to room-temperature or 4 °C cold stimulus, or a high-fat diet challenge as established protocols [48]. Briefly, place the chopped adipose tissue in a solution of type I collagenase and shake at 37 °C for 15 min to digest it. Then, add complete culture medium to halt digestion and filter sequentially through 100 μm and 70 μm cell strainers. Centrifuge the filtered solution at 300×g for 10 min. The white flocculent layer on top at this stage constitutes mature adipocytes and can be collected separately for subsequent Western blotting analysis. The pellet at the bottom is treated with erythrocyte lysis buffer for 5 min to yield SVF cells.

### Isolation of stromal vascular fraction (SVF) and differentiation into adipocytes

SVF cells were procured from the iWAT of male 6-week-old mice, as established protocols, with modifications for treatment and induction, and subsequently differentiated into mature adipocytes. Upon maturation, primary adipocytes underwent adenoviral infection for 48 h, then exposed to CL316,243 (2 µM, GC43276, GLPBIO) for 12 h, BAPTA-AM (1,2-bis(o-aminophenoxy) ethane-N, N,N’,N’-tetraacetic acid acetoxymethyl ester) (10 µM, GC13517, GLPBIO) for 2 h, KN93 (10 µM, HY-15465, MCE) for 12 h, Thapsigargin (1 µM, HY-13433, MCE) for 12 h, Carboxyatractyloside (CAT) (1 µM, HY-N1502, MCE) for 12 h or MI (50 µM, HY-B1411, MCE) for 24 h.

### Assays for IP3 and MI

Following maturation, primary adipocytes were infected with adenovirus for 48 h, gently washed, digested with trypsin, and obtained through at 1000 × g for 5 minutes. The collected cells were lysed by repeated freezing and thawing or sonication. Cell extracts were subjected to centrifugation at 1500 × g for 10 min at 4 °C, and the supernatant was utilized for IP3 assay *via* an ELISA kit (Elabscience, China) per the supplier’s protocols. For inositol assays, adipocytes were harvested at 5 × 10^5^ cells per 100 µL of assay buffer. After centrifugation of the cell homogenates at 10,000 × g for 5 minutes at 4 °C, the supernatants were collected for MI assay, following the manufacturer’s instructions (Abcam, USA).

### Ca^2+^ imaging

Primary adipocytes were plated onto collagen-coated glass bottom cell culture dishes (Rat Tail, cat. no. 40125ES10, YEASEN). Cells were incubated with 3 µM Fura-2AM (F0888, Sigma-Aldrich) in Krebs-Ringer Bicarbonate HEPES Buffer for 1 h at 37 °C per the supplier’s protocols. Afterward, the cells underwent triple washing using a normal Ca²⁺-containing solution in a 37 °C water bath. Fura-2AM-labeled adipocytes were maintained in HEPES Buffer for 30 s before being exposed to 1 µM norepinephrine (NE, GC30744, GLPBIO). Intracellular Ca²⁺ signals were recorded and calibrated as previously described [49], with a total recording duration of 300 s.

### Histological analysis of tissues

For hematoxylin-eosin (H&E) staining, iWAT, BAT, eWAT, and liver specimens underwent fixation in 4% paraformaldehyde at 4 °C overnight, embedded in paraffin, and sectioned for histological analysis. The tissue slices received H&E staining. For immunohistochemical analysis, the tissue segments underwent epitope retrieval in 10 mM sodium citrate buffer (pH 6.0) at 100 °C, then blocking with goat serum at room temperature for 1 h. The sections were exposed to primary antibodies targeting F4/80 (Proteintech, 28463-1-AP, 1:4000), UCP1 (Abcam, ab10983, 1:200) or IMPA2 (Sigma-Aldrich, HPA029561, 1:200) overnight at 4 °C, and subsequently developed using a DAB kit (P0203, Beyotime). For Oil Red O staining, the specimens were rapidly frozen, sectioned using a cryostat, and then stained. An Olympus microscope (Japan) was utilized to obtain images.

### Immunoblotting

Protein extraction from cells and tissues was performed utilizing RIPA buffer containing protease inhibitor cocktail (HY-K0010, MCE). Cell and tissue debris were removed by centrifugation at 12,000 rpm for 15 min at 4 °C After determination of the protein concentration with a standard bicinchoninic acid assay kit (Thermo Scientific), 20 µg of protein was electrophoresed and transferred to PVDF membranes, and blocking with 5% nonfat skim milk at room temperature for 1 h. The membranes were exposed to primary antibodies against IMPA2 (Abcam, ab256410, 1:1000), UCP1 (Abcam, ab10983, 1:1000), CamKIIα (Proteintech, 13730-1-AP, 1:1000), p-CaMKIIα (Santa cruz, sc-32289, 1:1000), PGC1α (Abclonal, A20995, 1:1000), PPARδ (Proteintech, 28053-1-AP, 1:1000), IRS1 (Proteintech, 17509-1-AP, 1:1000), P-IRS1 (Proteintech, 85238-1-RR, 1:1000), AKT (Proteintech, 10176-2-AP, 1:1000), P-AKT (Proteintech, 28731-1-AP, 1:1000), TFAM (Proteintech, 22586-1-AP, 1:1000), MTCO1 (Boster, BA2149, 1:1000) and β-actin (Abclonal, AC004, 1:2000) overnight at 4 °C on a horizontal shaker. A Clinx imaging system (Shanghai, China) was utilized to detect protein signals, and ImgeJ was employed to quantify band density.

### RNA extraction and quantitative real-time PCR

Total RNA was procured from adipose tissues and cells utilizing TRIzol reagent. The extracted RNA samples underwent reverse transcription utilizing the Reverse Transcription Kit (R323-01, Vazyme). Real-time PCR (qPCR) was executed utilizing SYBR Master Mix (Q511-02, Vazyme) on the ABI Prism Step-One platform. The primer sequences are depicted in Table S1.

### Untargeted metabolomics assays

LC-MS grade acetonitrile (ACN) was purchased from Fisher Scientific (Loughborough, UK). Formic acid was obtained from TCI (Shanghai, China). Ammonium formate was obtained from Sigma-Aldrich (Shanghai, China). Ultrapure water was generated using a Milli-Q system (Millipore, Bedford, USA). Adipocytes were trypsinized and counted (10^7^). Transfer adipocytes samples into a 2 mL centrifuge tube, add 100 mg glass bead. Add 1 mL acetonitrile: methanol: H_2_O mixed solution (2:2:1, V/V/V), vortex for 30 s. The samples were rapidly frozen in liquid nitrogen for 5 min, and when thawed, the samples were placed in a tissue grinder and ground at 60 Hz for 2 min. Repeat the liquid nitrogen freezing and grinding steps twice. The sample was centrifuged at 12,000 rpm for 10 min at 4℃, and the supernatant was concentrated and dried; 300 µL of 2-chloro-L-phenylalanine (4 ppm) prepared with acetonitrile: 0.1% formic acid (1:9, V/V) was added to redissolve the sample, and the supernatant was filtered through a 0.22 μm membrane, and the filtered solution was added into the assay vials for LC-MS detection.

### Untargeted metabolomics analysis and RNA-Seq analysis of NCBI GEO Datasets

The raw data of untargeted metabolomics were firstly converted to mzXML format by MSConvert in ProteoWizard software package (v3.0.8789) and processed using R XCMS (v3.12.0). Differential metabolites in control and overexpressing IMPA2 groups were ascertained utilizing the “limma” R package (version 3.54.2). By default, when P-value < 0.05 and and VIP value > 1, we think that metabolite was considered to have significant differential expression.

Differentially expressed genes between room temperature and cold-exposed iWAT samples in the GSE86338 and GSE164219 datasets were ascertained utilizing the “limma” R package (version 3.54.2). A gene was considered differentially expressed if it met the criteria: adjusted P-value < 0.05 and |log2 fold change| ≥ 1.0.

### Quantification of mtDNA copy number

The levels of NADH dehydrogenase subunit 1 (Nd1, mtDNA) were quantified (using qPCR) and normalized to levels of LPL (genomic DNA). The primer sequences for mt-Nd1 and LPL are depicted in Table S1.

### Seahorse

Approximately 10^4^ SVF cells were plated in each well of XF24 cell culture microplates (Agilent, 100777-004). When the adipocytes are differentiated and mature, the mature adipocytes were washed three times with XF24 medium (Agilent, 103575-100). After a 1 h pre-incubation in XF24 medium at 37 °C without CO_2_ in incubator, oxygen consumption rate (OCR) was measured using the XF Extracellular Analyzer (Agilent, 102340-100). The compounds used to determine OCR included 2 µM oligomycin, 0.5 µM carbonyl cyanide-p-trifluoromethoxyphenylhydrazone (FCCP), 2 µM antimycin A and 0.5 µM rotenone. The OCR value were normalized with protein concentration after the experiment was completed.

### Statistical analysis

Data are denoted as mean ± SEM. Statistical evaluations were executed utilizing GraphPad Prism 8.3 (GraphPad Software, USA). Comparisons between pairs of groups were evaluated through unpaired two-tailed Student’s t-test. For analyzing multiple groups, one-way or two-way ANOVA with subsequent Tukey’s post-hoc analysis was employed. Statistical significance was defined as *P* < 0.05. Cell experiments were performed in three independent replicates.

## Results

### IMPA2 level in iWAT of mice is upregulated by thermogenic stimulation and downregulated by obesity

**Fig. 1:**
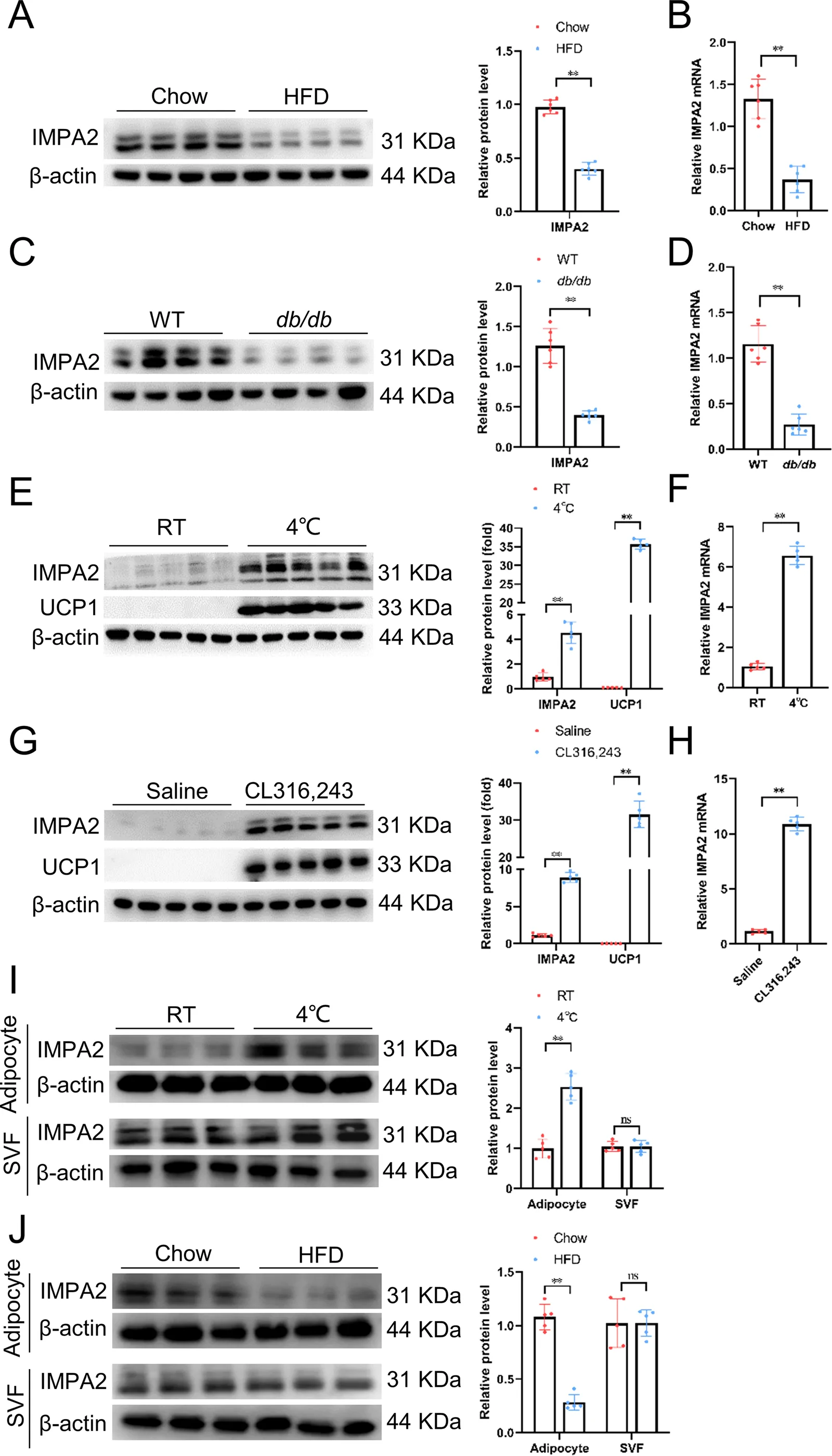
IMPA2 expression is upregulated by thermogenic stimulation and downregulated in obesity. Representative immunoblotting images of the expression levels of IMPA2 in iWAT from HFD-fed mice (**A**) for 12 weeks or db/db mice (**C**), Right panel shows the result of densitometric analyses. Relative mRNA levels of IMPA2 in iWAT from HFD-fed mice (**B**) or db/db mice (**D**). (**E**-**F**) Mice were maintained at room temperature (RT, 30 °C) or in a cold environment (4 °C) for 48 h, and iWAT was collected for further analysis. (**E**) Representative immunoblotting images of the expression levels of IMPA2, UCP1, and β-actin in iWAT. Right panel shows the result of densitometric analyses. (**F**) Relative mRNA levels of IMPA2. (**G**-**H**) Mice were injected intraperitoneally daily with saline or CL316,243 (1 mg/kg/d) for 5 d, and iWAT was collected for further analysis. (**G**) Representative immunoblotting images of the expression levels of IMPA2, UCP1, and β-actin in iWAT. Right panel shows the result of densitometric analyses. (**H**) Relative mRNA levels oIMPA2. Data represent mean ± SEM (*n* = 6). (**I**) Representative immunoblots and quantification of IMPA2 expression in SVF cells and adipocytes from iWAT under cold stimulation (4 °C). Right panel shows the result of densitometric analyses. (**J**) Representative immunoblots and quantification of IMPA2 expression in SVF cells and adipocytes from iWAT under chow or HFD feeding. Right panel shows the result of densitometric analyses. *P < 0.05, **P < 0.01. ns, non-significant

A systematic analysis was conducted on the publicly available cold-stimulus-induced expression datasets GSE86338 and GSE164219, revealing 120 overlapping upregulated genes (Fig. S1A), with IMPA2 demonstrating a significant increase in both datasets (Fig. S1B). To elucidate the role of IMPA2 in iWAT browning, its expression in obesity models and under thermogenic stimulation was examined. In iWAT from HFD-fed mice (Fig. 1A, B) and db/db mice (Fig. 1C, D), both protein and mRNA levels of IMPA2 were notably diminished. The role of IMPA2 in thermogenesis was further evaluated. The findings revealed notably enhanced IMPA2 levels in iWAT both under cold exposure (Fig. 1E, F, and S1C) and after CL316,243 treatment (Fig. 1G, H, and S1D). To identify the cell type responsible for IMPA2 expression under cold exposure and the cell type in which IMPA2 is predominantly downregulated in obesity, we isolated SVF cells and mature adipocytes from iWAT in both mouse models. We found that changes in IMPA2 protein levels were mainly observed in mature adipocytes rather than SVF cells (Fig. 1I, J).

However, IMPA2 levels remained unchanged in brown adipose tissue (BAT) and epididymal white adipose tissue (eWAT) under cold exposure (Fig. S2A-D) or CL316,243 administration (Fig. S2E-H). These observations collectively indicate that IMPA2 serves a crucial function in iWAT browning processes.

### Overexpression of IMPA2 in iWAT prevents HFD-induced obesity by promoting energy expenditure

**Fig. 2:**
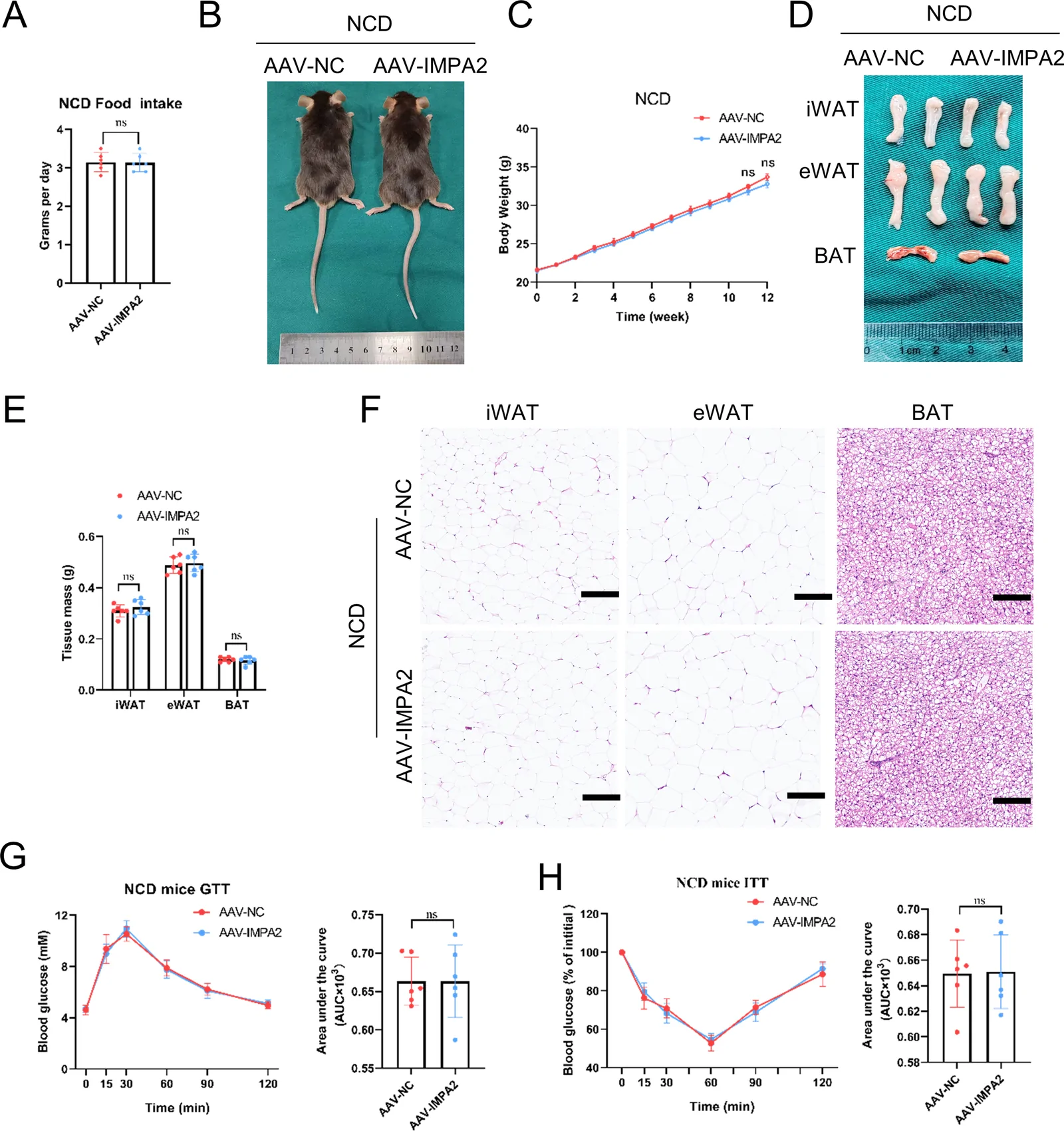
IMPA2 overexpression in iWAT prevents HFD-induced obesity by promoting energy expenditure. AAV-NC or AAV-IMPA2 was subcutaneously injected into the bilateral inguinal areas, and 2 weeks later, the mice received 12 weeks of the HFD. (**A**) Food intake of mice with injection of AAV-NC or AAV-IMPA2 (*n* = 6). (**B**) Gross morphology of mice before sacrifice. (**C**) Body weight of the mice (*n* = 6). (**D**) Representative images of BAT, iWAT, and eWAT from AAV-IMPA2-treated and AAV-NC-treated HFD mice. (**E**) Representative H&E staining of adipose tissues (iWAT, eWAT, and BAT) after 12 weeks of the HFD. Scale bar, 100 μm. (**F**) Adipose tissue (iWAT, eWAT, and BAT) weights of mice (*n* = 6). (**G**) Morphometric analysis of adipocytes. VO_2_ (**H**), VCO_2_ (**I**), and heat production (**J**) of mice were analyzed after 12 weeks of the HFD (*n* = 4). The day/night bar represents a 12-h duration. (**K**) Quantitative analysis of VO_2_ in panel H. (**L**) Quantitative analysis of VCO_2_ in panel I. (**M**) Quantitative analysis of heat production in panel J. (**N**) Average values of physical activity. VO_2_, VCO_2_, and heat production were analyzed by ANOVA with total body mass as a covariate (*n* = 4). (**O**) Representative IHC staining images of F4/80 in eWAT. Scale bar, 100 μm. The right panel shows the percentage area positive for F4/80. (**P**) Representative Oil Red O staining images of skeletal muscle sections. Scale bar, 100 μm. Data represent mean ± SEM. *P < 0.05. **P < 0.01. ns, non-significant

Additional investigations examined IMPA2’s influence on HFD-induced obesity. Subcutaneous adipose received either recombinant AAV-NC or AAV-IMPA2, followed by 12 weeks of HFD or a normal chow diet (NCD) administration. During the NCD feeding period, weight increase and food consumption remained equivalent between groups (Fig. S3A-C). Similarly, no significant differences were observed in adipose tissues weights, morphological assessments of adipose tissues (iWAT, eWAT, BAT), glucose tolerance, or insulin resistance between groups (Fig. S3D-H). Enhanced IMPA2 expression diminished HFD-induced weight increase, while food consumption remained equivalent between groups (Fig. 2A-C). Moreover, IMPA2 enhancement decreased iWAT and eWAT adipocytes size in obese conditions, while BAT stayed constant (Fig. 2D-F). Microscopic evaluation showed notably reduced adipocytes size in iWAT and eWAT (Fig. 2G). Considering equivalent food intake between groups (Fig. 2A), researchers proposed that IMPA2 might combat obesity through enhanced energy utilization. CLAMS evaluations confirmed that elevated IMPA2 expression resulted in greater oxygen uptake (VO_2_), carbon dioxide output (VCO_2_), and thermal generation compared to HFD-treated controls (Fig. 2H-M). Physical activity levels showed no significant variation (Fig. 2N). Importantly, immunohistochemical analysis revealed reduced macrophage infiltration in eWAT from IMPA2‑overexpressing mice, which was associated with improved inflammatory status and enhanced adaptive thermogenesis (Fig. 2O). Meanwhile, IMPA2‑overexpressing mice also exhibited decreased fatty infiltration in skeletal muscle (Fig. 2P).

### IMPA2 overexpression protects mice against obesity-induced metabolic dysfunction

**Fig. 3:**
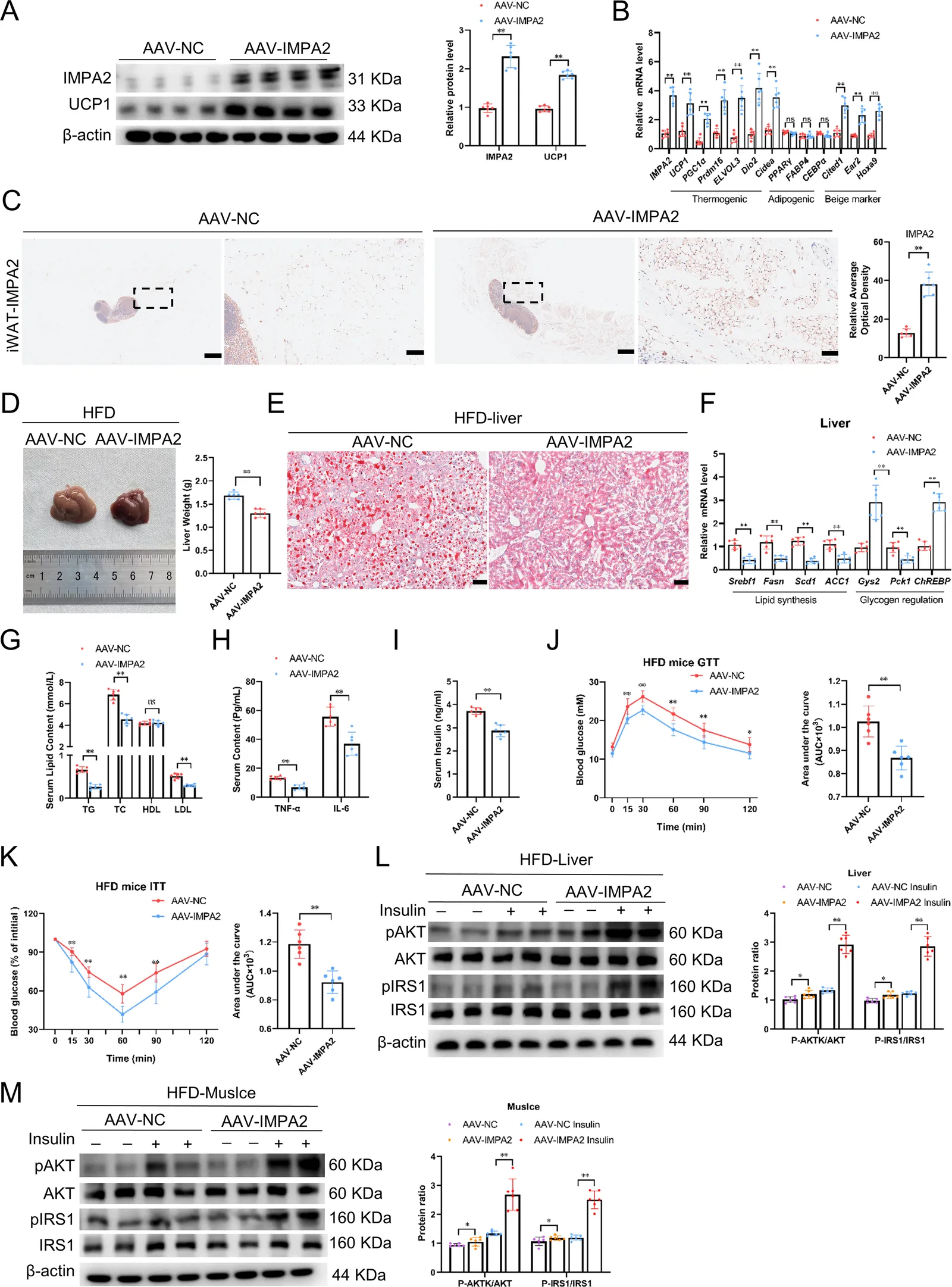
IMPA2 overexpression in iWAT protects mice against obesity-induced metabolic disorder. (**A**) Immunoblotting analysis of IMPA2 and UCP1 protein levels in iWAT. The right panel shows densitometric quantification. (**B**) Relative mRNA levels of thermogenic, adipogenic, and beige adipocyte marker genes. (**C**) Representative IHC staining images of IMPA2 in iWAT. Scale bar, 1 mm (left), 200 μm (right). Right panel shows quantitative analysis of IMPA2 immunohistochemical intensity. (**D**) Representative macroscopic images of liver tissues from AAV-IMPA2 and AAV-NC mice after 12 weeks of HFD feeding (*n* = 6). The right panel shows liver weights. (**E**) Representative Oil Red O staining images of liver sections. Scale bar, 100 μm. (**F**) Relative mRNA expression levels of genes involved in hepatic lipid synthesis and glycogen metabolism. (**G**) Serum levels of TG, TC, HDL, and LDL in mice (*n* = 6). (**H**) Serum levels of TNF-α and IL-6 in mice (*n* = 6). (**I**) Serum insulin levels in mice. (**J**) GTT in HFD-fed AAV-IMPA2 and AAV-NC mice (*n* = 6). The right panel shows the AUC of GTT. (**K**) ITT in HFD-fed AAV-IMPA2 and AAV-NC mice (*n* = 6). The right panel shows the AUC of ITT. (**L**) Representative immunoblots and quantification of p-AKT and p-IRS1 in liver tissues from HFD-fed AAV-IMPA2 and AAV-NC mice 10 min after intravenous injection of saline or insulin (1.5 unit/kg). (**M**) Representative immunoblots and quantification of p-AKT and p-IRS1 in skeletal muscle from HFD-fed AAV-IMPA2 and AAV-NC mice 10 min after intravenous injection of saline or insulin (1.5 unit/kg). Data are presented as mean ± SEM. *P < 0.05, **P < 0.01. ns, not significant

Subsequent examination revealed that enhanced IMPA2 expression elevated UCP1 protein levels in iWAT (Fig. 3A). Furthermore, thermogenic gene transcripts (*Ucp1*, *PGC1α*, *Prdm16*, *Elovl3*, *Dio2*, and *Cidea*) and beige indicator genes (*Cited1*, *Ear2*, and *Hoxa9*) showed significant upregulation with increased IMPA2 expression in HFD-treated subjects, while adipogenic gene expression (*PPARγ*, *Fabp4*, and *Cebpα*) remained stable (Fig. 3B). These findings indicate that elevated IMPA2 in iWAT mitigates HFD-induced obesity through enhanced energy utilization.

Obesity is widely recognized for its significant contribution to the development of metabolic disorders. Gross morphological analysis revealed that IMPA2 overexpression in iWAT notably reduced liver size and weight (Fig. 3D), while Oil Red O staining demonstrated reduced lipid accumulation in the liver (Fig. 3E). Furthermore, mRNA levels of genes involved in hepatic lipogenesis and glycogen synthesis were downregulated in the IMPA2‑overexpression group, whereas those related to gluconeogenesis were upregulated (Fig. 3F). Additional metabolic disorder–related markers, including serum TG, TC, HDL, LDL, circulating inflammatory cytokines, fasting serum insulin, glucose tolerance, and insulin sensitivity, were impaired in HFD-fed mice but significantly improved by IMPA2 overexpression (Fig. 3G-K). Furthermore, compared with HFD-fed control mice, higher levels of phosphorylated AKT (p-AKT) and phosphorylated IRS1 (p-IRS1) proteins were detected in the liver and skeletal muscle of HFD-fed, IMPA2-overexpressing mice treated with insulin (Fig. 3L, M), indicating improved insulin sensitivity. Together, these findings suggest that enhanced IMPA2 level in iWAT confers protection against obesity-induced metabolic dysfunction.

### Overexpression of IMPA2 promotes adaptive thermogenesis

**Fig. 4:**
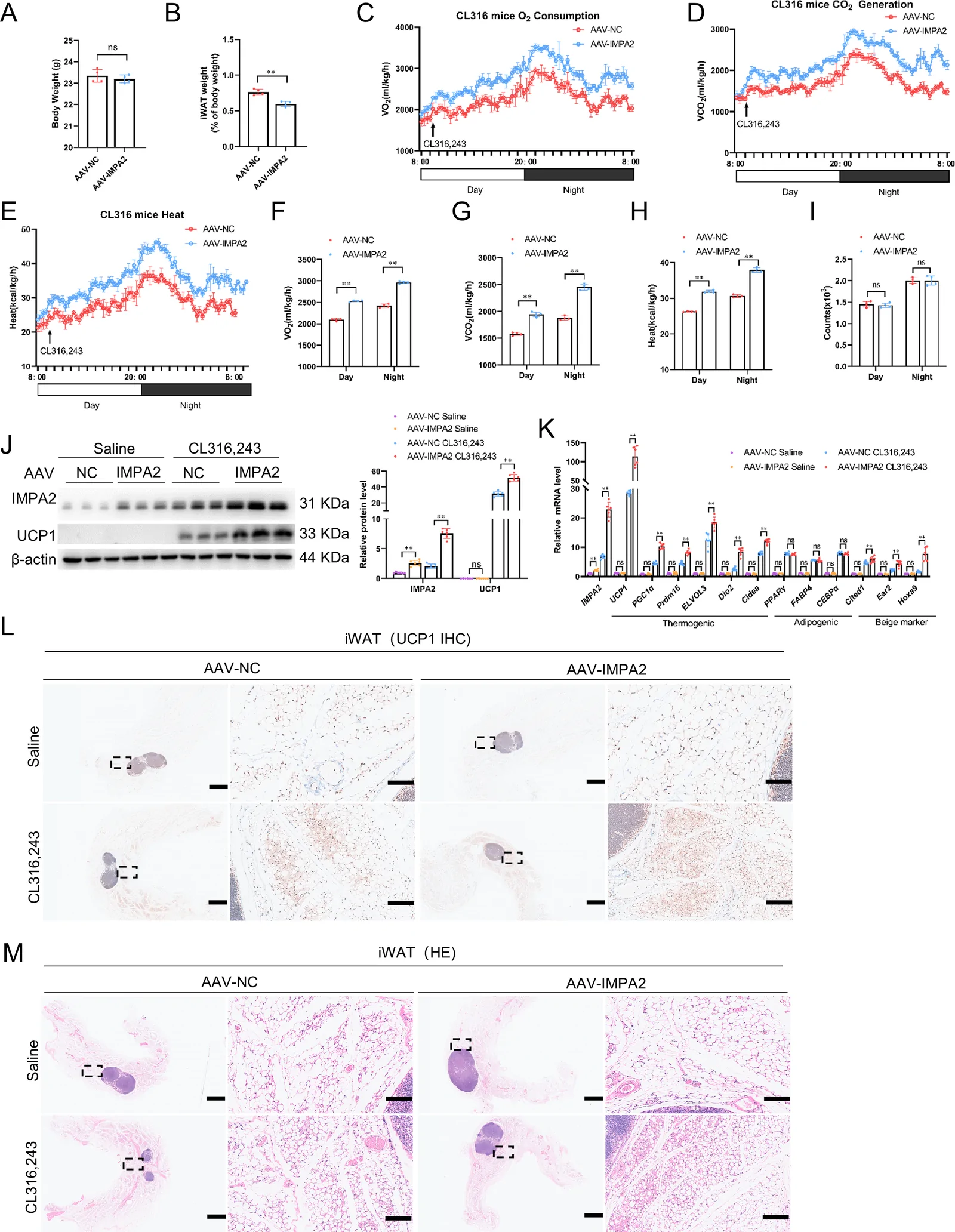
IMPA2 gain-of-function promotes thermogenic stimulation-induced browning of iWAT. AAV-NC or AAV-IMPA2 was subcutaneously injected into the bilateral inguinal areas, and 2 weeks later, the mice were subjected to cold exposure (4 °C) for 48 h before sacrifice. (**A**) The rectal temperature of mice was monitored in the first 6 h (*n* = 4). VO_2_ (**B**), VCO_2_ (**C**), and heat production (**D**) were analyzed (*n* = 4). The day/night bar represents a 12-h duration. (**E**) Quantitative analysis of VO_2_ in panel B. (**F**) Quantitative analysis of VCO_2_ in panel C. (**G**) Quantitative analysis of heat production in panel D. (**H**) Average values of physical activity (*n* = 4). (**I**) Representative immunoblotting images of the expression levels of IMPA2 and UCP1 in iWAT. Right panel shows the result of densitometric analyses. (**J**) Relative mRNA levels of thermogenic, adipogenic, and beige marker genes. (**K**) Images show UCP1 immunohistochemistry in iWAT sections; scale bar, 1 mm (left), 100 μm (right). (**L**) Representative H&E staining of iWAT sections; scale bar, 1 mm (left), 100 μm (right). VO_2_, VCO_2_, and heat production were analyzed by ANOVA with total body mass as a covariate. Data represent mean ± SEM. *P < 0.05, **P < 0.01. ns, non-significant

Given that IMPA2 enhances energy expenditure in HFD-fed mice, its influence on iWAT browning and adaptive thermogenesis was further examined using a cold-exposure model. Gain-of-function of IMPA2 was achieved through AAV-IMPA2 viral transduction, leading to efficient and specific expression in iWAT (Fig. 4I, J and S4J, L). IMPA2 overexpression improved cold tolerance at 4 °C and increased VO_2_, VCO_2_, and heat production compared to AAV-NC mice (Fig. 4A-G). However, no significant changes in locomotor activity were observed following IMPA2 intervention (Fig. 4H). There was no statistically significant difference in body weight between the two groups (Fig. S4A). In comparison to their controls, a significant decrease in iWAT mass was detected in the overexpression of IMPA2 group (Fig. S4B). Further examination of IMPA2 expression and genes related to adipose thermogenesis revealed that IMPA2 overexpression increased UCP1 expression in iWAT (Fig. 4I-K). In parallel, the mRNA levels of thermogenic genes and beige marker genes were upregulated in cold-exposed mice, whereas adipogenic gene levels remained unchanged (Fig. 4J). Moreover, IMPA2 overexpression in iWAT resulted in smaller adipocytes (Fig. 4K, L, and S4K). In contrast, under room temperature (30 °C), IMPA2 overexpression had no effect on VO_2_, VCO_2_, heat production, locomotor activity (Fig. S4C-I), thermogenic gene expression, or adipocyte size (Fig. 4J-L). In summary, cold exposure promoted IMPA2 expression (Fig. 1E, F), and elevated IMPA2 enhanced the adaptive thermogenesis response to cold (Fig. 4). These results suggest that IMPA2 serves as an effective accelerator of iWAT browning and adaptive thermogenesis.

The role of IMPA2 in iWAT browning was further explored using a β3-adrenoceptor agonist model, another key model for inducing browning. There was no statistically significant difference in body weight between the two groups (Fig. S5A). In comparison to their controls, a significant decrease in iWAT mass was detected in the overexpression of IMPA2 group (Fig. S5B). Consistent with the cold exposure model, AAV-IMPA2 treated mice exhibited increased VO_2_, elevated VCO_2_, and enhanced heat production compared to AAV-NC treated mice (Fig. S5C-H). However, locomotor activity remained unaffected (Fig. S5I). In vivo, IMPA2 overexpression induced by CL316,243 treatment resulted in reduced adipocyte size (Fig. S5L, M and S6A, B) and increased UCP1 protein levels (Fig. S5J, L), along with upregulation of thermogenic genes and beige marker genes, whereas adipogenic gene expression remained unchanged (Fig. S5K). In vitro, primary adipocytes were treated with CL316,243 and infected with Adenovirus-IMPA2 (Adv-IMPA2) to achieve efficient IMPA2 overexpression. This treatment significantly increased UCP1 protein levels and the mRNA expression of thermogenic genes (Fig. S6D, E). In summary, elevated IMPA2 expression enhanced metabolic rate and facilitated iWAT browning, indicating IMPA2’s potential as a therapeutic target for addressing obesity-related metabolic complications.

### Knockdown of IMPA2 inhibits adaptive thermogenesis

**Fig. 5:**
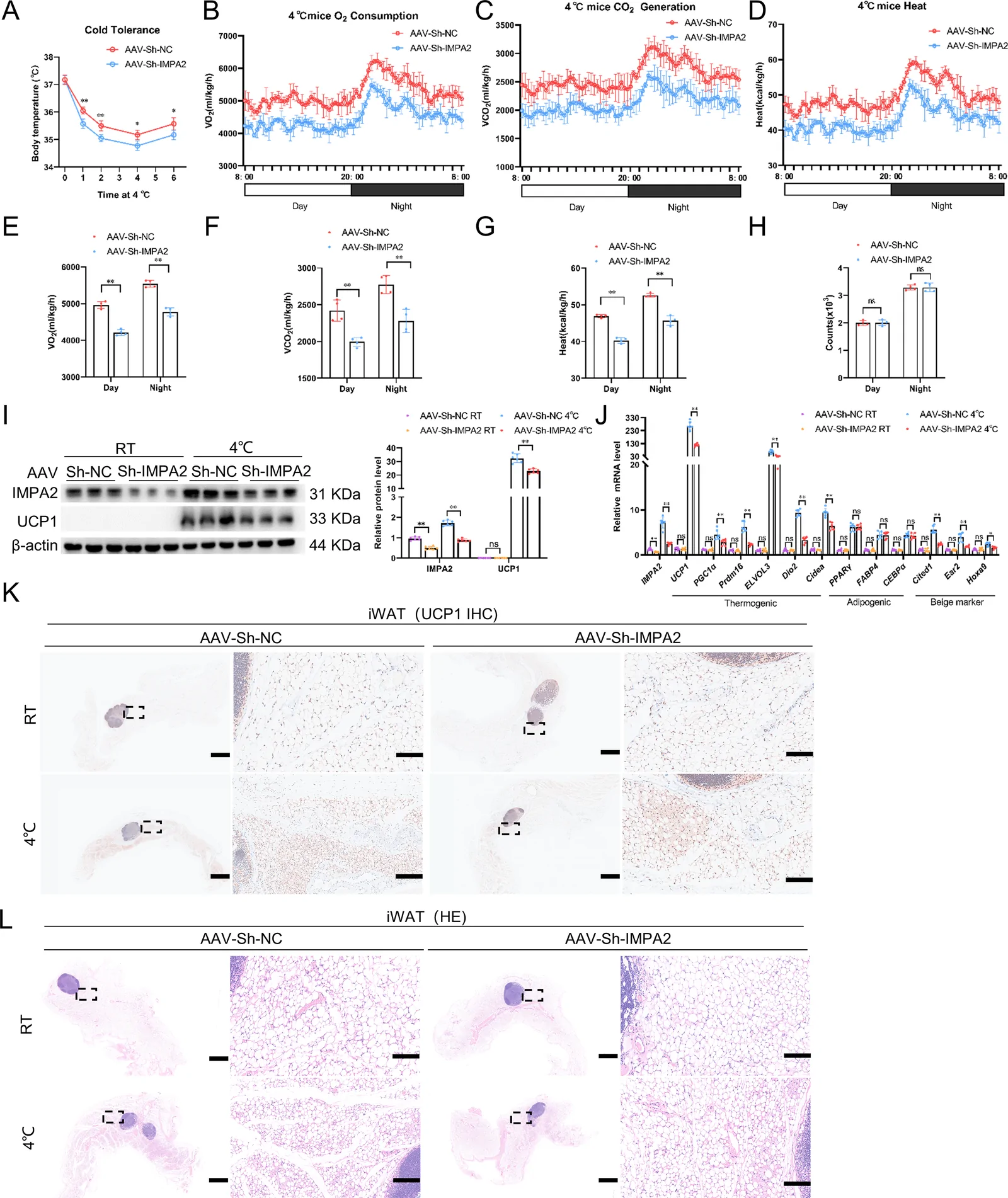
IMPA2 loss-of-function inhibits cold expose-induced browning of iWAT. AAV-Sh-NC or AAV-Sh-IMPA2 was subcutaneously injected into the bilateral inguinal areas, and 2 weeks later, the mice were subjected to cold exposure (4 °C) for 48 h before sacrifice. (**A**) The rectal temperature of mice was monitored in the first 6 h (*n* = 4). VO_2_ (**B**), VCO_2_ (**C**), and heat production (**D**) were analyzed (*n* = 4). The day/night bar represents a 12-h duration. (**E**) Quantitative analysis of VO_2_ in panel B. (**F**) Quantitative analysis of VCO_2_ in panel C. (**G**) Quantitative analysis of heat production in panel D. (**H**) Average values of physical activity (*n* = 4). VO_2_, VCO_2_, and heat production were analyzed by ANOVA with total body mass as a covariate. (**I**) Representative immunoblotting images of the expression levels of IMPA2 and UCP1 in iWAT. Right panel shows the result of densitometric analyses. (**J**) Relative mRNA levels of thermogenic, adipogenic, and beige marker genes. (**K**) Images show UCP1 immunohistochemistry in iWAT sections; scale bar, 1 mm (left), 100 μm (right). (**L**) Representative H&E staining of iWAT sections; scale bar, 1 mm (left), 100 μm (right). Data represent mean ± SEM. *P < 0.05, **P < 0.01. ns, non-significant

The potential of IMPA2 knockdown in iWAT to induce the opposite phenotype was further evaluated. Efficient IMPA2 knockdown was achieved using AAV-sh-IMPA2, as confirmed by immunoblotting, immunohistochemistry, and qPCR (Fig. 5I, J and S7J, L). Upon cold exposure, AAV-sh-IMPA2 mice exhibited compromised cold tolerance, reduced VO_2_, diminished VCO_2_, and lower heat production relative to AAV-sh-NC controls (Fig. 5A-G). Although AAV‑sh‑IMPA2 mice exhibited slightly less weight loss, the difference was not statistically significant (Fig. S7A). AAV‑sh‑IMPA2 mice also showed a smaller reduction in iWAT mass relative to control mice (Fig. S7B). No significant changes were detected in locomotor activity during cold exposure (Fig. 5H) or in basal metabolic rate at room temperature (Fig. S7C-I). Histological analyses, including immunohistochemistry and H&E staining, revealed a reduced number of multilocular adipocytes (Fig. 5K, L and Fig. S7J, K). Additionally, IMPA2 silencing in iWAT diminished cold-induced UCP1 (Fig. 5I, K). Consistent with these observations, the expression of thermogenic genes and beige marker genes was significantly downregulated, whereas adipogenic gene expression remained unaffected under cold exposure (Fig. 5J).

In the CL316,243 model, although AAV-sh-IMPA2 mice had slightly less weight loss, it was not statistically significant (Fig. S8A). The iWAT mass of AAV-sh-IMPA2 mice decreased less compared to the control group (Fig. S8B). Knockdown of IMPA2 in iWAT resulted in marked reductions in VO_2_, VCO_2_, and heat production compared to the AAV-sh-NC control group (Fig. S8C-H), while locomotor activity (Fig. S8I) showed no significant differences. As with cold exposure, IMPA2 silencing suppressed UCP1 expression (Fig. S8J-L and S9D, E) and thermogenic gene levels as well as beige marker genes, without affecting the expression of adipogenic genes induced by CL316,243 (Fig. S8K). Furthermore, the size of multilocular adipocytes were reduced (Fig. S8L, M and S9A, B). To explore the role of IMPA2 in white-to-beige adipocyte conversion, primary adipocytes derived from the SVF of iWAT from WT mice were used in vitro. CL316,243 treatment elevated UCP1 protein levels and mRNA expression of thermogenic markers in cells, effects that were attenuated by IMPA2 silencing (Fig. S9D, E) but amplified by IMPA2 overexpression (Fig. S6D, E). These results collectively suggest that IMPA2 facilitates white-to-beige adipocyte conversion, thereby promoting adaptive thermogenesis. Additional investigations are required to thoroughly characterize the precise thermogenic mechanisms through which IMPA2 modulates adaptive thermogenesis.

### IMPA2 regulates the thermogenic function of adipocytes in a MI-IP3-Ca²⁺-dependent pathway

**Fig. 6:**
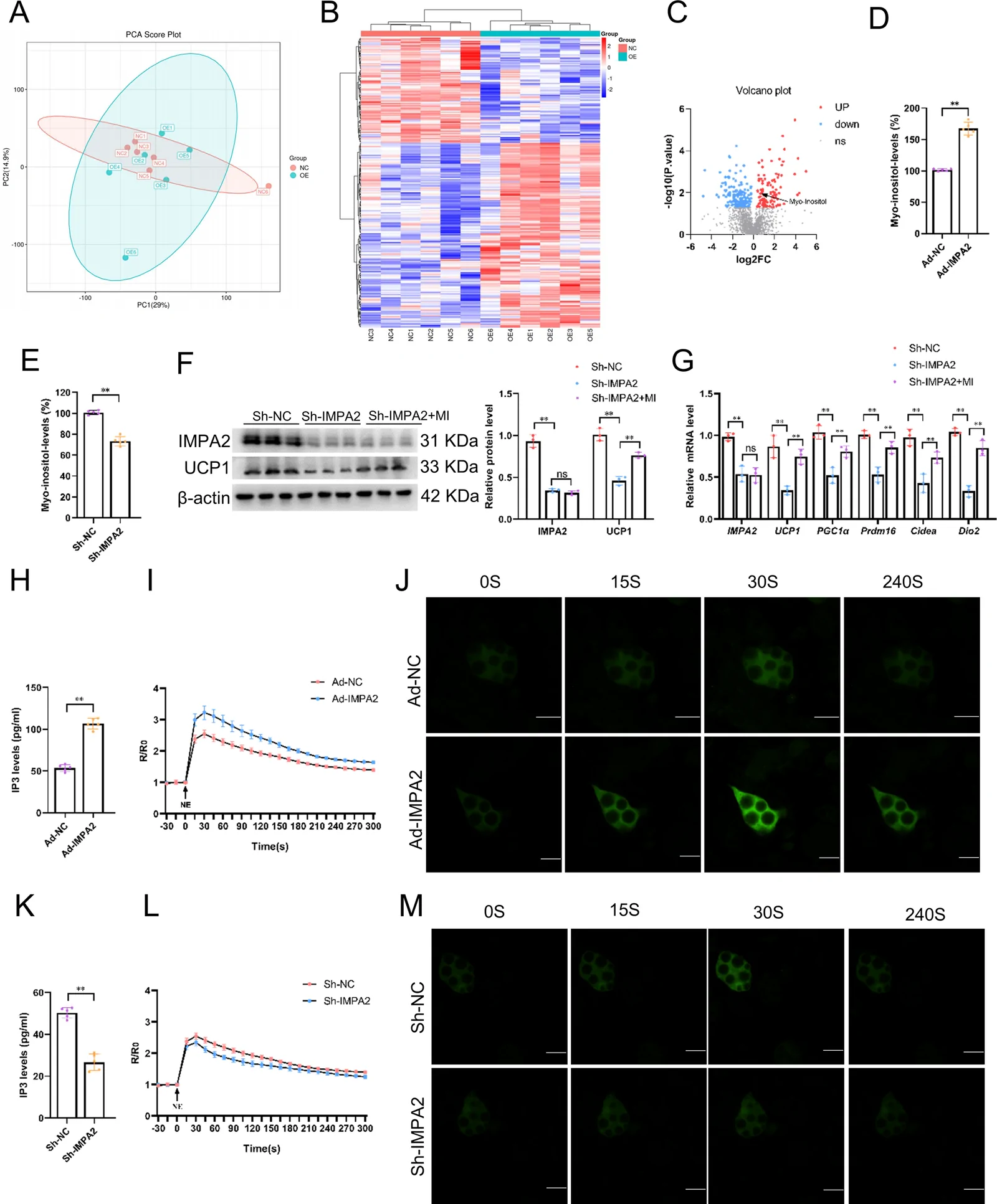
IMPA2 regulates thermogenic function of brown adipocytes in a MI-IP3-Ca^2+^-dependent pathway. Primary adipose cells were treated with Ad-NC or Ad-IMPA2 (**D**, **H**-**J**), or Sh-NC or Sh-IMPA2 (**E**-**G**, **K**-**M**). (**A**) PCA model score plot of the untargeted metabolomics. (**B**) Heat map of differential metabolite clustering. (**C**) Volcano plot of differential metabolites. (**D**) Myo-inositol levels of primary adipose cells treated with Ad-NC or Ad-IMPA2. (**E**) Myo-inositol levels of primary adipose cells treated with Sh-NC or Sh-IMPA2. (**F**) Representative immunoblotting images of the expression levels of IMPA2 and UCP1 in primary adipose cells treated with Sh-NC, Sh-IMPA2 or MI (50 µM) treated sh-IMPA2 after exposing to 100-mOsm sucrose for 10 min. Right panel shows the result of densitometric analyses. (**G**) Relative mRNA levels of IMPA2 and thermogenic genes. (**H**) IP3 levels was examined by ELISA in primary adipose cells treated with Ad-NC or Ad-IMPA2. (**I**) Quantitative analysis of the Fura2-AM fluorescence intensity (R/R0) of primary adipose cells treated with Ad-NC or Ad-IMPA2 following exposure to 1 µM NE. (**J**) Representative time-lapse images of indicated primary adipose cells at different time intervals after 1 µM NE stimulation; scale bar, 50 μm. (**K**) IP3 levels were examined by ELISA in primary adipose cells treated with Sh-NC or Sh-IMPA2. (**L**) Quantitative analysis of the Fura2-AM fluorescence intensity (R/R0) of primary adipose cells treated with Sh-NC or Sh-IMPA2 following exposure to 1 µM NE. (**M**) Representative time-lapse images of indicated primary adipose cells at different time intervals after 1 µM NE stimulation; scale bar, 50 μm. Data represent mean ± SEM. *P < 0.05, **P < 0.01. ns, non-significant

Untargeted metabolomics techniques were used to explore the mechanisms by which IMPA2 promotes thermogenesis in adipocytes. When adipocytes were induced to differentiate and mature, IMPA2 overexpressing cells and control cells were sent for untargeted metabolomics assay. As can be seen from the PCA score plots (Fig. 6A), all samples were within 95% confidence intervals, using untargeted metabolomics (R2X = 0.574). Metabolomic analysis identified a total of 2166 metabolites, of which 96 were upregulated and 167 were downregulated. Among the upregulated metabolites, myo-inositol—a downstream metabolite of IMPA2—was significantly upregulated (Fig. 6B, C). These results indicate that myo‑inositol may play a critical role in IMPA2‑mediated promotion of the adipocyte thermogenic pathway. We subsequently measured inositol levels in IMPA2‑overexpressing adipocytes using an ELISA kit. Myo‑inositol concentrations were significantly increased upon IMPA2 overexpression (Fig. 6D), which were reduced upon IMPA2 knockdown (Fig. 6E). Furthermore, overexpression of IMPA2 in SVF cells also led to an increase in inositol levels compared with the control group, indicating the widespread nature of this pathway (Fig. S10A). In vivo, local overexpression of IMPA2 in iWAT resulted in increased inositol levels within the iWAT, but did not lead to an increase in serum inositol levels compared with the control group (Fig. S10B, C). To enhance inositol uptake, adipocytes were pre‑exposed to 100‑mOsm sucrose for 10 min. Supplementation with MI rescued UCP1 expression that had been suppressed by IMPA2 silencing (Fig. 6F). In addition, the relative mRNA levels of thermogenic genes downregulated by IMPA2 knockdown were restored by MI supplementation (Fig. 6G). Previous studies have shown that the IMPA2 enzyme binds Ca²⁺ [22]. Additionally, IMPA2 has been implicated in elevating intracellular Ca²⁺ concentration by promoting the MI-PI cycle and IP3 production in BLBC cells [29]. As reported, Ca²⁺ serves a critical function in adipocyte thermogenesis [28–31]. Based on this, it was hypothesized that IMPA2 may modulate thermogenic programs through an MI-IP3-Ca²⁺ signaling pathway. Therefore, IP3 levels in adipocytes were assessed. Consistent with MI levels, IP3 levels were elevated in Ad-IMPA2-treated cells (Fig. 6H) and reduced in Sh-IMPA2-treated cells (Fig. 6K). To delineate IMPA2’s regulation of Ca²⁺ homeostasis, we performed live-cell Ca²⁺ imaging with Fura-2 AM to quantify NE-induced intracellular Ca²⁺ fluxes in adipocytes. IMPA2 overexpression increased intracellular Ca²⁺ levels (Fig. 6I, J), while IMPA2 knockdown resulted in reduced Ca²⁺ density (Fig. 6L, M), indicating that IMPA2 regulates Ca²⁺ signaling in adipocytes.

### IMPA2 regulates the thermogenic function of adipocytes in a Ca²⁺-mediated mitochondrial biogenesis pathway

**Fig. 7:**
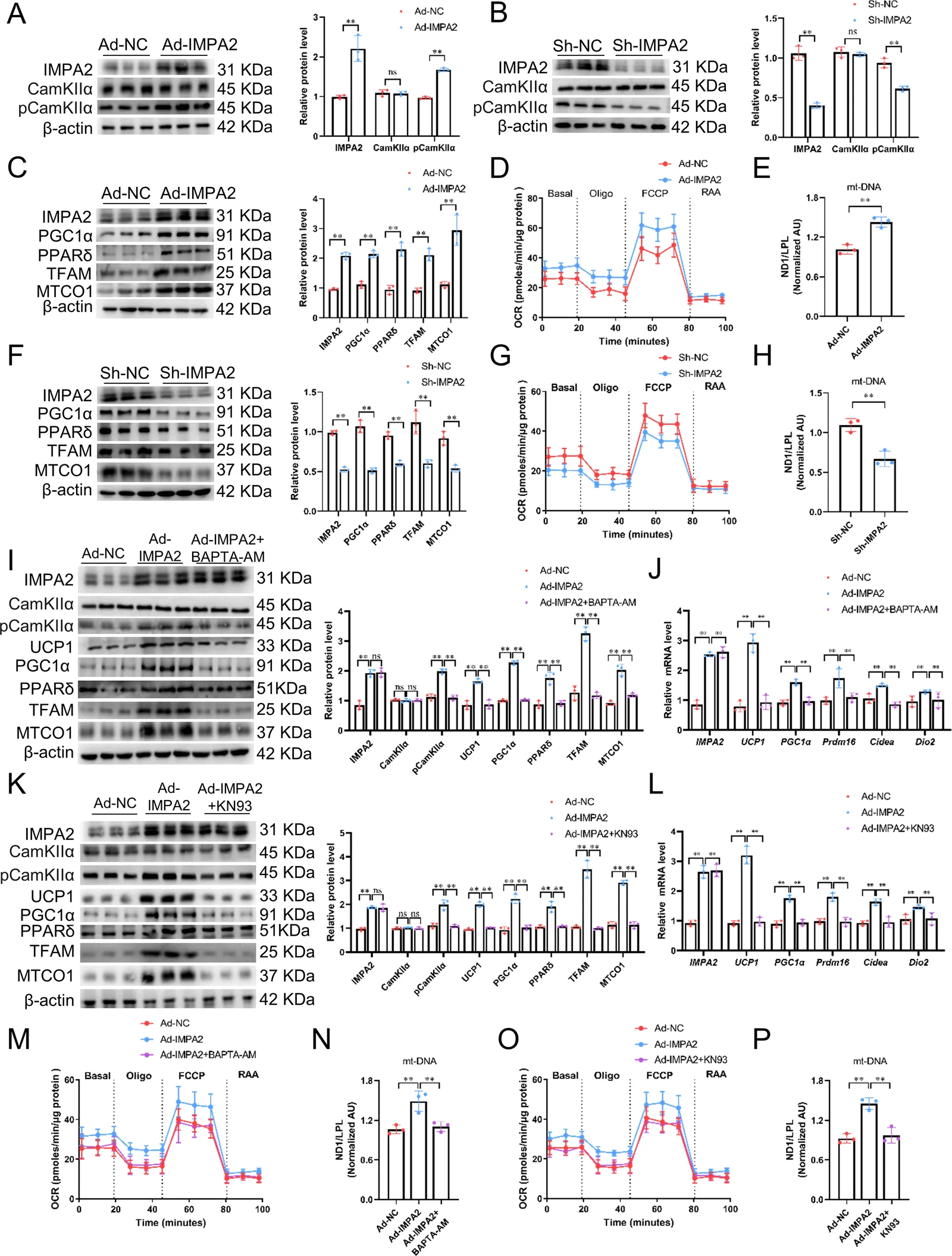
Overexpression of IMPA2 promotes increased mitochondrial biogenesis in adipocytes depending on activation of CamKIIα. Primary adipose cells were treated with Ad-NC or Ad-IMPA2 (**A**, **C**-**E**, **I**-**P**), or Sh-NC or Sh-IMPA2 (**B**, **F**-**H**). (**A**, **B**) Representative immunoblotting images of the expression levels of IMPA2, CamKIIα and p-CamKIIα. Right panel shows the result of densitometric analyses. (**C**, **F**) Representative immunoblotting images of the expression levels of IMPA2 and mitochondrial biogenesis indicators. Right panel shows the result of densitometric analyses. (**D**, **G**) OCR in primary adipose cells. (**E**, **H**) MtDNA content in primary adipose cells. (**I**-**J**, **M**-**N**) Primary adipose cells were treated with Ad-NC, Ad-IMPA2, or Ad-IMPA2 plus 10 µM BAPTA‑AM for 2 h. (**I**) Representative immunoblotting images of the expression levels of IMPA2, UCP1, CamKIIα, p-CamKIIα and mitochondrial biogenesis indicators in primary adipose cells treated. Right panel shows the result of densitometric analyses. (**J**) Relative mRNA levels of IMPA2 and thermogenic genes. (**M**) OCR in primary adipose cells. (**N**) MtDNA content in primary adipose cells. (**K**-**L**, **O**-**P**) Primary adipose cells were treated with Ad-NC, Ad-IMPA2 or Ad-IMPA2 plus 10 µM KN93 for 12 h. (**K**) Representative immunoblotting images of the expression levels of IMPA2, UCP1, CamKIIα, p-CamKIIα and mitochondrial biogenesis indicators in primary adipose cells treated. Right panel shows the result of densitometric analyses. (**L**) Relative mRNA levels of IMPA2 and thermogenic genes. (**O**) OCR in primary adipose cells. (**P**) MtDNA content in primary adipose cells. Data represent mean ± SEM. *P < 0.05, **P < 0.01. ns, non-significant

Previous studies have shown that elevated intracellular Ca²⁺ levels activate phosphorylated calcium/calmodulin-dependent protein kinase IIα (p-CaMKIIα), which promotes mitochondrial biogenesis in skeletal muscle [45]. These findings led us to hypothesize that IMPA2 regulates thermogenesis through a Ca²⁺-dependent mitochondrial biogenesis mechanism. Next, CamKIIα and p-CamKIIα in adipocytes were assessed. Protein levels of p-CamKIIα were significantly elevated by IMPA2 overexpression (Fig. 7A) and reduced by IMPA2 knockdown (Fig. 7B). Adipocytes overexpressing IMPA2 exhibited elevated levels of mitochondrial biogenesis–related proteins (including PGC1α, PPARδ, TFAM, and MTCO1), as well as increased OCR and mitochondrial content (Fig. 7C-E). In contrast, IMPA2 knockdown significantly reduced mitochondrial biogenesis–related proteins, OCR, and mitochondrial content (Fig. 7F-H). Similarly, in vivo experiments also showed the same trend in mitochondrial content (Fig. S10D-H). To determine whether IMPA2-mediated white-to-beige adipocyte reprogramming and mitochondrial biogenesis are Ca²⁺-dependent, we used BAPTA-AM, a membrane-permeable Ca²⁺ chelator. Immunoblotting results demonstrated that BAPTA-AM attenuated IMPA2-induced iWAT browning. Specifically, the increased expression levels of thermogenic genes (*Ucp1*, *PGC1α*, *Prdm16*, *Cidea*, and *Dio2*), as well as the elevated levels of thermogenic and mitochondrial biogenesis-related proteins (including UCP1, PGC1α, TFAM, and PPARδ) induced by IMPA2 upregulation, were partially attenuated by BAPTA-AM treatment (Fig. 7I, J). Furthermore, BAPTA-AM treatment blocked IMPA2-induced CaMKIIα phosphorylation in adipocytes, consequently attenuating the increases in mitochondrial biogenesis and respiratory capacity (Fig. 7I, M, N). Recent studies have reported that the SERCA2b-dependent Ca²⁺ cycle is one of the mechanisms underlying UCP1-independent thermogenesis [14, 16]. We investigated whether IMPA2 also promotes thermogenesis in adipocytes via UCP1-independent pathway. We added the SERCA2b inhibitor thapsigargin to IMPA2-overexpressing adipocytes; however, the thapsigargin did not block or significantly attenuate the increase in oxygen consumption induced by IMPA2 overexpression (Fig. S10I). To determine whether IMPA2 enhances mitochondrial function, mitochondrial biogenesis, or both, we treated adipocyte with inositol for either 4–48 h. The results indicated that the 4-hour treatment did not increase mitochondrial copy number or improve oxygen consumption, whereas the 48-hour treatment was accompanied by an increase in both mitochondrial copy number and oxygen consumption (Fig. S10J, K). Furthermore, IMPA2 did not affect changes in genes associated with mitochondrial fusion, fission and autophagy (Fig. S10L). To determine whether IMPA2-mediated white-to-beige adipocyte reprogramming and mitochondrial biogenesis is CamKIIα-dependent, KN93, a CamKIIα specific inhibitors, was employed. Results showed that CamKIIα-activated mitochondrial biogenesis and respiratory capacity as well as the expression of thermogenic genes were inhibited by pretreatment with KN93 (Fig. 7K, L, O, P). Since the adenine nucleotide translocator (ANT) can also contribute to proton leak leading to thermogenesis [50], we also used the ANT inhibitor CAT to investigate whether IMPA2 promotes thermogenesis in adipocytes *via* the ANT pathway. The results confirmed that CAT did not inhibit the increase in oxygen consumption induced by IMPA2 overexpression (Fig. S10M). Collectively, these results suggest that IMPA2 regulates adaptive thermogenesis *via* Ca²⁺-mediated mitochondrial biogenesis pathway.

## Discussion

Enhancing energy expenditure by promoting iWAT browning is considered one of the promising future strategies for preventing obesity [2]. While IMPA2 has been extensively studied for its role as a tumor-related gene marker, its function in obesity remains poorly understood. This study specifically investigates the metabolic role of IMPA2 signaling in thermogenic adipocytes.

Adaptive (non-shivering) thermogenesis is a critical component of energy expenditure [51]. Previous reports have shown that cold stimulation activates several signaling pathways, including MAPK, insulin, and Ca²⁺ signaling pathways [52]. For instance, Ca²⁺ supplementation has been shown to enhance the activation and thermogenesis of BAT and the browning of WAT in HFD-fed mice [53]. Ca²⁺ are involved in both UCP1-independent and UCP1-dependent adipose thermogenesis. Beige adipocytes possess ATP-dependent, UCP1-independent Ca²⁺ cycling machinery to dissipate energy as heat [54]. As dynamic organelles, mitochondria are central to adipocyte energy metabolism, primarily through ATP production *via* oxidative phosphorylation [55]. Mitochondrial dysfunction has been directly or indirectly linked to the origin of many diseases such as obesity and type 2 diabetes. Obesity has been shown to be associated with reduced mitochondrial respiration, increased mitochondrial ROS production, and dysregulated mitochondrial biogenesis [56, 57]. Collectively, these findings position mitochondrial function restoration as a translatable therapeutic approach for metabolic dysfunction in obesity.

**Fig. 8:**
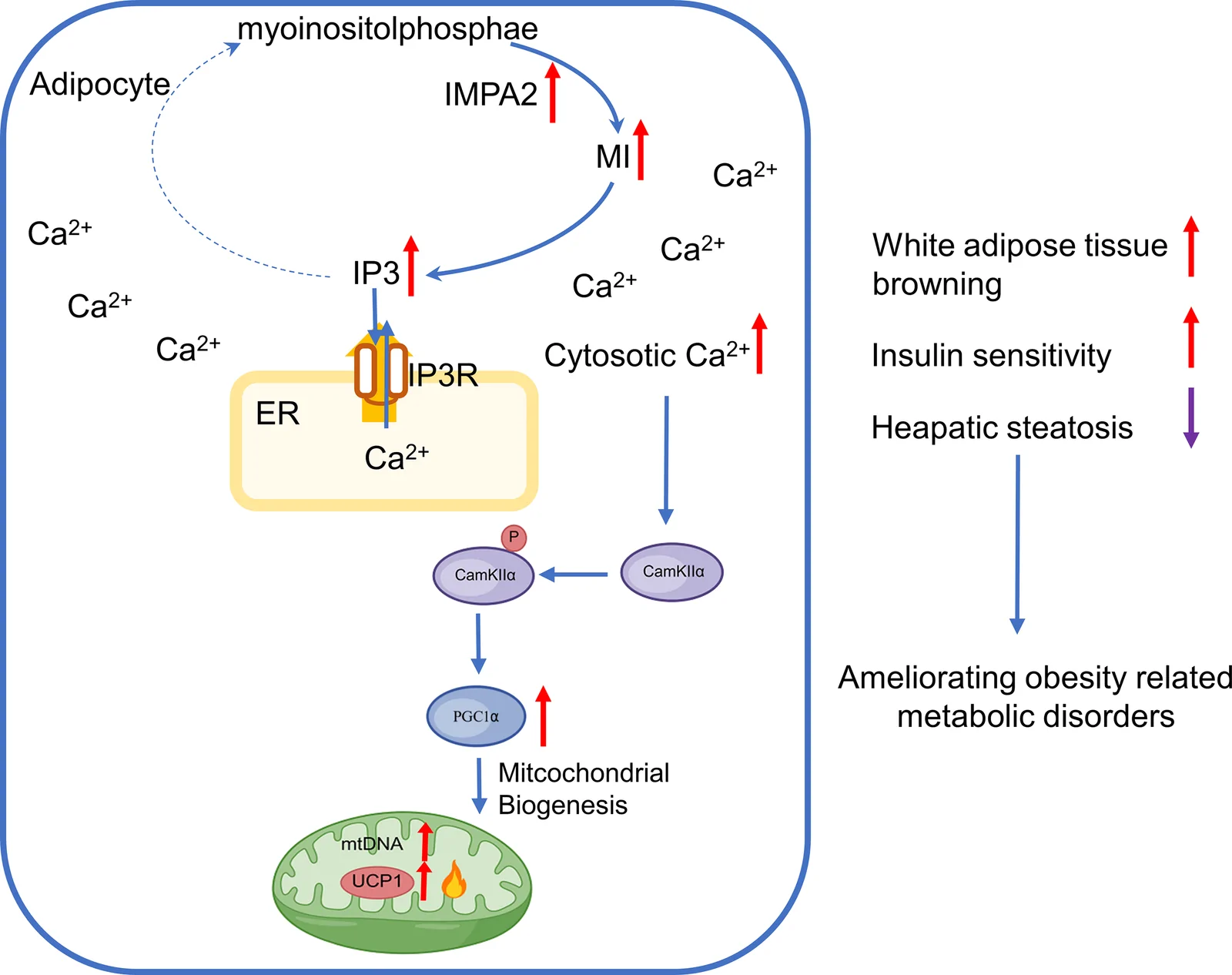
Schematic diagram of IMPA2-mediated adaptive thermogenesis via Ca²⁺-dependent mitochondrial biogenesis

This study demonstrates that IMPA2 acts as a positive regulator of beige adipocyte thermogenesis, thereby influencing systemic glucose homeostasis. IMPA2 overexpression in adipocytes enhanced WAT browning capacity, which conferred protection against HFD-induced glucose intolerance, insulin resistance, and liver steatosis. The metabolic benefits correlated with increased whole-body energy expenditure and reduced weight gain. In contrast, mice with adipocyte-specific ablation of IMPA2 showed reduced thermogenesis and impaired energy expenditure. Mechanistically, IMPA2 regulates the thermogenic function of adipocytes in a Ca²⁺-mediated mitochondrial biogenesis pathway. Given IMPA2’s enzymatic function, we hypothesized it regulates adipocyte metabolites. Metabolomic analysis revealed IMPA2 overexpression induced widespread metabolic reprogramming (96 upregulated and 167 downregulated metabolites), particularly demonstrating a dramatic elevation in inositol levels. We then hypothesized that overexpression of IMPA2 catalyzes the conversion of myo-inositol-1-phosphate to MI, which leads to the formation of IP3 and a subsequent rise in intracellular Ca²⁺ concentration. Consistent with our hypothesis, ELISA and live-cell Ca²⁺ imaging confirmed that IMPA2 overexpression elevated intracellular Ca²⁺ levels. This triggered Ca²⁺-dependent signaling pathways, primarily through CaMKIIα activation and subsequent PGC1α upregulation. The observed cytoplasmic Ca²⁺ transients promoted mitochondrial biogenesis *via* PGC1α-mediated transcriptional activation rather than UCP1-independent thermogenic pathway involving SERCA2b-dependent calcium cycling and ANT causes proton leak. Pharmacological inhibition with BAPTA-AM and KN93 revealed that IMPA2-mediated PGC1α activation requires both Ca²⁺ signaling and CaMKIIα activity (Fig. 8). Our data suggest that IMPA2 promotes iWAT browning by enhancing Ca²⁺ signaling and augmenting mitochondrial function in adipocytes, thereby coordinating energy demand with increased energy production.

In conclusion, this study uncovers a previously unrecognized IMPA2-Ca²⁺-activated mitochondrial biogenesis pathway that regulates iWAT browning. This discovery provides new insights into the pathogenesis of obesity-related metabolic disorders and highlights potential therapeutic targets for their treatment. Collectively, our study emphasizes the critical role of IMPA2 in regulating beige adipocyte thermogenesis and systemic glucose homeostasis, revealing a novel pathway that could be leveraged for treating obesity and associated metabolic disorders.

## Supplementary Information

Below is the link to the electronic supplementary material.

Supplementary Material 1

Supplementary Material 2

Supplementary Material 3

Supplementary Material 4

Supplementary Material 5

Supplementary Material 6

Supplementary Material 7

Supplementary Material 8

Supplementary Material 9

Supplementary Material 10

Supplementary Material 11

Supplementary Material 12

## Data Availability

The data sets supporting the conclusions of this article are included within the article.
