# Supplementary material for: IMPA2 ameliorates high-fat diet-induced obesity by enhancing adaptive thermogenesis in a Ca²⁺-activated mitochondrial biogenesis pathway
Source: J Transl Med. 2026 Jul 8;24:1238. doi: 10.1186/s12967-026-08592-2 (PMC13632368; doi:10.1186/s12967-026-08592-2)
Supplement: Supplementary file 1 [file 12967_2026_8592_MOESM11_ESM.docx]

Table S1 Primers used in this study

| The name of the gene | Primer sequences（5'→3'） |
| --- | --- |
| IMPA2 F | TGCGTGCGGGACAAATCAT |
| IMPA2 R | GAAACCGCTTTCGCAACTCAG |
| 18S F | GCACCACCACCCACGGAATCG |
| 18S R | TTGACGGAAGGGCACCACCAG |
| UCP1 F | TGCCAGGCAAGCTGAAACTC |
| UCP1 R | GTGAACCCGACAACTTCCGAA |
| PGC1α F | TATGGAGTGACATAGAGTGTGCT |
| PGC1α R | GTCGCTACACCACTTCAATCC |
| Prdm16 F | TGCTGACGGATACAGAGGTGT |
| Prdm16 R | CCACGCAGAACTTCTCGCTAC |
| ELOVL3 F | TTCTCACGCGGGTTAAAAATGG |
| ELOVL3 R | TCTCGAAGTCATAGGGTTGCAT |
| CEBPα F | TTCGGGTCGCTGGATCTCTA |
| CEBPα R | CCCGAGAGGAAGCAGGAATC |
| Dio2 F | CAGTGTGGTGCACGTCTCCAATC |
| Dio2 R | TGAACCAAAGTTGACCACCAG |
| Cidea F | TGCTCTTCTGTATCGCCCAGT |
| Cidea R | CGGTGCTAACACGACAGGG |
| FABP4 F | AAGGTGAAGAGCATCATAACCCT |
| FABP4 R | TCACGCCTTTCATAACACATTCC |
| PPARγ F | GGAAGACCACTCGCATTCCTT |
| PPARγ R | GTAATCAGCAACCATTGGGTCA |
| Cited1 F | AACCTTGGAGTGAAGGATCGC |
| Cited1 R | GTAGGAGAGCCTATTGGAGATGT |
| Ear2 F | TCCAGGACAACCAGCCCTAA |
| Ear2 R | GTGTTCTCCTCCGACTGGTGA |
| Hoxa9 F | CCCCGACTTCAGTCCTTGC |
| Hoxa9 R | GATGCACGTAGGGGTGGTG |
| mt-Nd1 F | CCCATTCGCGTTATTCTT |
| mt-Nd1 R | AAGTTGATCGTAACGGAAGC |
| LPL F | GGATGGACGGTAAGAGTGATTC |
| LPL R | ATCCAAGGGTAGCAGACAGGT |
| Srebf1 F | TGACCCGGCTATTCCGTGA |
| Srebf1 R | CTGGGCTGAGCAATACAGTTC |
| FASN F | GGAGGTGGTGATAGCCGGTAT |
| FASN R | TGGGTAATCCATAGAGCCCAG |
| SCD1 F | TTCTTGCGATACACTCTGGTGC |
| SCD1 R | CGGGATTGAATGTTCTTGTCGT |
| ACC1 F | CTCCCGATTCATAATTGGGTCTG |
| ACC1 R | TCGACCTTGTTTTACTAGGTGC |
| GYS2 F | CGCTCCTTGTCGGTGACATC |
| GYS2 R | CATCGGCTGTCGTTTTGGC |
| PCK1 F | CTGCATAACGGTCTGGACTTC |
| PCK1 R | GCCTTCCACGAACTTCCTCAC |
| ChREBP F | AGATGGAGAACCGACGTATCA |
| ChREBP R | AGATGGAGAACCGACGTATCA |
| MFN1 F | ATGGCAGAAACGGTATCTCCA |
| MFN1 R | GCCCTCAGTAACAAACTCCAGT |
| OPA1 F | TGGAAAATGGTTCGAGAGTCAG |
| OPA1 R | CATTCCGTCTCTAGGTTAAAGCG |
| DRP1 F | CAGGAATTGTTACGGTTCCCTAA |
| DRP1 R | CCTGAATTAACTTGTCCCGTGA |
| FIS1 F | AGAGCACGCAATTTGAATATGCC |
| FIS1 R | ATAGTCCCGCTGTTCCTCTTT |
| PINK1 F | TGCTGAAACTGCCTTCCTATCA |
| PINK1 R | CCGCTAGTTGAACATACAGGATG |
| LC3B F | TTATAGAGCGATACAAGGGGGAG |
| LC3B R | CGCCGTCTGATTATCTTGATGAG |
| P62 F | ACAGCCCAAACGTGCAGTAA |
| P62 R | CTGATGCGGAACTACATCTGAAT |
