# Supplementary material for: IMPA2 ameliorates high-fat diet-induced obesity by enhancing adaptive thermogenesis in a Ca²⁺-activated mitochondrial biogenesis pathway
Source: J Transl Med. 2026 Jul 8;24:1238. doi: 10.1186/s12967-026-08592-2 (PMC13632368; doi:10.1186/s12967-026-08592-2)
Supplement: Supplementary file 2 [file 12967_2026_8592_MOESM12_ESM.docx]

Figure S1 IMPA2 expression in public transcriptomic datasets and immunohistochemical validation. (A) Venn diagram of upregulated genes in adipose tissue stimulated by cold exposure (GSE86338 and GSE164219). (B) Box plots indicated IMPA2 mRNA expression of iWAT at RT and 4 ℃ from GSE86338 and GSE164219 datasets. (C) Immunohistochemical analysis of IMPA2 staining under indicated conditions. Scale bar, 1 mm (left), 100 μm (right). Right panel shows the result of densitometric analyses. (D) Immunohistochemical analysis of IMPA2 staining under indicated conditions. Scale bar, 1 mm (left), 100 μm (right). Right panel shows the result of densitometric analyses.

Figure S2 IMPA2 expression in BAT and eWAT induced by thermogenic stimulation. (A-D) Mice were maintained at room temperature (RT, 30 ℃) or in a cold environment (4 ℃) for 48 h, and BAT and eWAT were collected for further analysis. (A) Representative immunoblotting images of the expression levels of IMPA2, UCP1, and β-actin in BAT. Right panel shows the result of densitometric analyses. (B) Relative mRNA levels of UCP1 and IMPA2 in BAT. (C) Representative immunoblotting images of the expression levels of IMPA2, UCP1, and β-actin in eWAT. Right panel shows the result of densitometric analyses. (D) Relative mRNA levels of UCP1 and IMPA2 in eWAT. (E-H) Mice were injected intraperitoneally daily with saline or CL316,243 (1 mg/kg/d) for 5 d, and BAT and eWAT were collected for further analysis. (E) Representative immunoblotting images of the expression levels of IMPA2, UCP1, and β-actin in BAT. Right panel shows the result of densitometric analyses. (F) Relative mRNA levels of UCP1 and IMPA2 in BAT. (G) Representative immunoblotting images of the expression levels of IMPA2, UCP1, and β-actin in eWAT. Right panel shows the result of densitometric analyses. (H) Relative mRNA levels of UCP1 and IMPA2 in eWAT. Data represent mean ± SEM (n = 6). *P < 0.05, **P < 0.01. ns, non-significant. eWAT, epididymal WAT.

Figure S3 Adipose-specific overexpression of IMPA2 does not affect metabolic phenotypes under normal chow diet (NCD). (A) Food intake of mice with injection of AAV-NC or AAV-IMPA2 (n=6). (B) Gross morphology of mice before sacrifice. (C) Body weight of the mice (n=6). (D) Representative images of BAT, iWAT, and eWAT from NCD-fed mice treated with AAV-NC or AAV-IMPA2. (E) Adipose tissue (iWAT, eWAT, and BAT) weights of mice (n=6). (F) Representative H&E staining of adipose tissues (iWAT, eWAT, and BAT) after 12 weeks of NCD feeding. Scale bar, 100 μm. (G) Glucose tolerance test (GTT) and corresponding area under the curve (AUC) in NCD-fed mice (n=6). (H) Insulin tolerance test (ITT) and corresponding AUC in NCD-fed mice (n=6). Data represent mean ± SEM. *P < 0.05, **P < 0.01. ns, non-significant.

Figure S4 Adipose-specific overexpression of IMPA2 does not affect adipose thermogenesis at room temperature. AAV-NC or AAV-IMPA2 was subcutaneously injected into the bilateral inguinal regions. Two weeks later, mice were housed at room temperature or under cold exposure before sacrifice. (A) Body weight of mice under cold exposure. (B) Relative iWAT weight normalized to body weight under cold exposure. (C) VO₂, (D) VCO₂, and (E) heat production at room temperature were analyzed. The day/night bar represents a 12-h duration. (F) Quantitative analysis of VO_2_ shown in panel C. (G) Quantitative analysis of VCO_2_ shown in panel D. (H) Quantitative analysis of heat production shown in panel E. (I) Average values of physical activity. (J) Represented IHC staining images of IMPA2 in iWAT. Scale bar, 1 mm (left), 100 μm (right). (K) Morphometric analysis of adipocyte size. (L) Quantitative analysis of IMPA2 immunohistochemical intensity. VO_2_, VCO_2_, and heat production were analyzed by ANOVA with total body mass as a covariate. Data represent mean ± SEM (n = 4). *P < 0.05, **P < 0.01. ns, non-significant.

Figure S5 IMPA2 gain-of-function promotes adipose thermogenesis induced by CL316,243. AAV-NC or AAV-IMPA2 was subcutaneously injected into the bilateral inguinal areas, and 2 weeks later, the mice were injected intraperitoneally daily with saline or CL316,243 (1 mg/kg/d) for 5 d. VO_2_, VCO_2_, heat production of mice in response to CL316,243 treatment at day 5. (A) Body weight of the mice (n=4). (B) Relative iWAT weight (normalized to body weight) after CL316,243 treatment at day 5 (n=4). VO_2_ (C), VCO_2_ (D), and heat production (E) were analyzed (n=4). The day/night bar represents a 12-h duration. (F) Quantitative analysis of VO_2_ in panel C. (G) Quantitative analysis of VCO_2_ in panel D. (H) Quantitative analysis of heat production in panel E. (I) Average values of physical activity. (J) Representative immunoblotting of IMPA2 and UCP1 in iWAT. Right panel shows densitometric quantification. (K) Relative mRNA levels of thermogenic, adipogenic, and beige marker genes. (L) Immunohistochemical staining of UCP1 in iWAT. Scale bars: 1 mm (left), 100 μm (right). (M) Representative H&E staining of iWAT. Scale bars: 1 mm (left), 100 μm (right). VO_2_, VCO_2_, and heat production were analyzed by ANOVA with total body mass as a covariate. Data represent mean ± SEM. *P < 0.05, **P < 0.01. ns, non-significant.

Figure S6 IMPA2 gain-of-function promotes adipose thermogenesis induced by CL316,243 *in vivo and in vitro*. (A) Representative immunohistochemical staining of IMPA2 in iWAT. Scale bars: 1 mm (left), 100 μm (right). (B) Morphometric analysis of adipocyte size. (C) Quantitative analysis of IMPA2 immunohistochemical intensity. (D-E) Primary adipocytes were infected with Ad-NC or Ad-IMPA2 for 48 h, then treated with PBS or 2 μM CL316,243. (D) Representative immunoblots of IMPA2 and UCP1 in primary adipocytes. Right panel shows densitometric quantification. (E) Relative mRNA levels of *Impa2*, *Ucp1*, Pgc1α, *Prdm16*, *Cidea*, and *Dio2*. Data represent mean ± SEM. *P < 0.05, **P < 0.01. ns, non-significant.

Figure S7 Knockdown of IMPA2 does not affect adipose thermogenesis at room temperature. AAV‑sh‑NC or AAV‑sh‑IMPA2 was subcutaneously injected into the bilateral inguinal regions. Two weeks later, mice were housed at room temperature or under cold exposure before sacrifice. (A) Body weight of mice under cold condition. (B) Relative iWAT weight normalized to body weight under cold exposure. (C) VO₂, (D) VCO₂, and (E) heat production at room temperature were analyzed. Day/night bars indicate a 12‑hour cycle. (F) Quantification of VO₂ from panel C. (G) Quantification of VCO₂ from panel D. (H) Quantification of heat production from panel E. (I) Average physical activity. (J) Representative immunohistochemical staining of IMPA2 in iWAT. Scale bars: 1 mm (left), 100 μm (right). (K) Morphometric analysis of adipocyte size. (L) Quantitative analysis of IMPA2 immunohistochemical intensity. VO_2_, VCO_2_, and heat production were analyzed by ANOVA with total body mass as a covariate. Data represent mean ± SEM (n = 4). *P < 0.05, **P < 0.01. ns, non-significant.

Figure S8 Knockdown of IMPA2 inhibits CL316,243‑induced adipocyte thermogenesis. AAV‑sh‑NC or AAV‑sh‑IMPA2 was subcutaneously injected into the bilateral inguinal regions. Two weeks later, mice received daily intraperitoneal injections of saline or CL316,243 (1 mg/kg/d) for 5 days. VO₂, VCO₂, and heat production were measured on day 5 after CL316,243 administration. (A) Body weight of mice (n=4). (B) Relative iWAT weight normalized to body weight on day 5 after CL316,243 treatment (n=4). (C) VO₂, (D) VCO₂, and (E) heat production were analyzed (n=4). Day/night bars indicate a 12‑hour cycle. (F) Quantification of VO₂ from panel C. (G) Quantification of VCO₂ from panel D. (H) Quantification of heat production from panel E. (I) Average physical activity. (J) Representative immunoblots of IMPA2 and UCP1 in iWAT. Right panel shows densitometric quantification. (K) Relative mRNA levels of thermogenic, adipogenic, and beige marker genes. (L) Immunohistochemical staining of UCP1 in iWAT. Scale bars: 1 mm (left), 100 μm (right). (M) Representative H&E staining of iWAT. Scale bars: 1 mm (left), 100 μm (right). VO2, VCO2, and heat production were analyzed by ANOVA with total body mass as a covariate. Data represent mean ± SEM (n = 4). *P < 0.05, **P < 0.01. ns, non-significant.

Figure S9 Knockdown of IMPA2 inhibits CL316,243‑induced adipocyte thermogenesis *in vivo* and *in vitro*. (A) Representative immunohistochemical staining of IMPA2 in iWAT. Scale bars: 1 mm (left), 100 μm (right). (B) Morphometric analysis of adipocyte size. (C) Quantitative analysis of IMPA2 immunohistochemical intensity. (D-E) Primary adipocytes were transfected with Sh‑NC or Sh‑IMPA2 for 24 h, then treated with PBS or 2 μM CL316,243. (D) Representative immunoblots of IMPA2 and UCP1 in primary adipocytes. Right panel shows densitometric quantification. (E) Relative mRNA levels of *Impa2*, *Ucp1*, *Pgc1α*, *Prdm16*, *Cidea*, and *Dio2*. Data represent mean ± SEM (n = 4). *P < 0.05, **P < 0.01. ns, non-significant.

Figure S10 IMPA2 regulates myo‑inositol levels, mitochondrial DNA copy number, and respiratory function in adipocytes. (A) Myo‑inositol levels in SVF cells infected with Ad‑NC or Ad‑IMPA2. (B) Myo‑inositol levels in iWAT from mice treated with AAV‑NC or AAV‑IMPA2. (C) Serum myo‑inositol levels in mice treated with AAV‑NC or AAV‑IMPA2. (D) Mitochondrial DNA (mtDNA) content in iWAT from HFD‑fed mice treated with AAV‑NC or AAV‑IMPA2. (E) mtDNA content in iWAT from cold‑exposed mice treated with AAV‑NC or AAV‑IMPA2. (F) MtDNA content in iWAT from cold‑exposed mice treated with AAV‑Sh‑NC or AAV‑Sh‑IMPA2. (G) MtDNA content in iWAT from mice co‑treated with AAV‑NC or AAV‑IMPA2 and CL316,243. (H) MtDNA content in iWAT from mice co‑treated with AAV‑Sh‑NC or AAV‑Sh‑IMPA2 and CL316,243. (I) Oxygen consumption rate (OCR) in primary adipocytes infected with Ad‑NC, Ad‑IMPA2, or Ad‑IMPA2 and Thapsigargin (1 μM). (J) OCR in primary adipocytes treated with myo‑inositol (MI) for 4 h or 48 h. (K) MtDNA content in primary adipocytes treated with MI for 4 h or 48 h. (L) Relative mRNA levels of genes related to mitochondrial fusion, fission, and mitophagy. (M) OCR in primary adipocytes infected with Ad‑NC, Ad‑IMPA2, or Ad‑IMPA2 and CAT (1 μM). Data represent mean ± SEM (n = 4). *P < 0.05, **P < 0.01. ns, non-significant.
